# Identification of novel glycosyl hydrolases with cellulolytic activity against crystalline cellulose from metagenomic libraries constructed from bacterial enrichment cultures

**DOI:** 10.1186/2193-1801-3-365

**Published:** 2014-07-16

**Authors:** Toshio Mori, Ichiro Kamei, Hirofumi Hirai, Ryuichiro Kondo

**Affiliations:** Department of Agro-environmental Sciences, Faculty of Agriculture, Kyushu University, Fukuoka, 812-8581 Japan; Department of Forest and Environmental Sciences, Faculty of Agriculture, University of Miyazaki, Miyazaki, 889-2192 Japan; Department of Applied Biological Chemistry, Faculty of Agriculture, Shizuoka University, Shizuoka, 422-8529 Japan

**Keywords:** Metagenome, Glycosyl hydrolase, Cellulase, Xylanase

## Abstract

To obtain cellulases that are capable of degrading crystalline cellulose and cedar wood, metagenomic libraries were constructed from raw soil sample which was covered to pile of cedar wood sawdust or from its enrichment cultures. The efficiency of screening of metagenomic library was improved more than 3 times by repeating enrichment cultivation using crystalline cellulose as a carbon source, compared with the library constructed from raw soil. Four cellulase genes were obtained from the metagenomic libraries that were constructed from the total genome extracted from an enrichment culture that used crystalline cellulose as a carbon source. A cellulase gene and a xylanase gene were obtained from the enrichment culture that used unbleached kraft pulp as a carbon source. The culture supernatants of *Escherichia coli* expressing three clones that were derived from the enrichment culture that used crystalline cellulose showed activity against crystalline cellulose. In addition, these three enzyme solutions generated a reducing sugar from cedar wood powder. From these results, the construction of a metagenomic library from cultures that were repetition enriched using crystalline cellulose demonstrated that this technique is a powerful tool for obtaining cellulases that have activity toward crystalline cellulose.

**Electronic supplementary material:**

The online version of this article (doi:10.1186/2193-1801-3-365) contains supplementary material, which is available to authorized users.

## Background

Lignocellulosic pools are a renewable source of feedstock for biofuel production. Cellulose, hemicellulose, and lignin are closely associated, and covalent cross-linkages have been suggested to occur between lignin and polysaccharides. Cellulose is the most abundant biomass in nature. Cellulose has great potential for a number of applications, including biofuel production. The cost of ethanol production from lignocellulosic materials is high, and the main challenge is high cost of the hydrolysis process. Numerous studies have been performed to improve the hydrolysis of lignocelluloses by pretreatment (Sun and Cheng 2002).

Lignocellulosic biomass can be converted to ethanol by hydrolysis and downstream fermentation processing. This process is much more complicated than the fermentation of a C6 sugar, and is more expensive than the production of bioethanol from starch or sugar crops. Structural features of native biomass limit accessibility to enzymes or microorganisms. Lignocellulose is difficult to hydrolyze because it is associated with hemicellulose, it is surrounded by a lignin seal that has a limited covalent association with hemicellulose, and much of cellulose has a crystalline structure (Weil et al. 1994). Especially, depolymerization step is the rate-limiting step for the whole cellulose hydrolysis process.

Cellulolytic fungi and bacteria play an important role in carbon cycle in nature. Especially, filamentous fungi are main microbe performing cellulose degradation in aerobic environment. Several strains of *Trichoderma* produce an extracellular cellulase complex that degrades native cellulose (Wojtczak et al. 1987). Fungal cellulases act synergistically with endoglucanases (EC 3.2.1.4, hydrolyze internal β-1,4-glucosidic linkages randomly in the cellulose chain), exoglucanases (also known as cellobiohydorolases, breakdown celllose into cellobiose from the ends), and β-glucosidases (EC 3.2.1.21, hydrolyze cellobiose and cellooligosaccharides to glucose) for cellulosic hydrolysis. Many other fungi produce cellulases and degrade soluble cellulose derivatives such as carboxymethylcellulose (CMC). However, they are not so effective on crystalline cellulosic substrates. Although mesophilic fungal strains produce cellulase, these fungal cellulases have limited efficiency in cellulose hydrolysis (Kumar et al. 2008). Some bacterial strains that produce cellulases are also able to degrade cellulose in aerobic or facultative anaerobic conditions (e.g. Kato et al. 2005).

Bacterial cellulases have the advantages over fungus for the genetic improvement and for the economic production because there are technical difficulties of construction of enzyme expression system from fungal species. Although many of cellulases are already characterized, a further screenings of new cellulases that have better characteristics (strong activity, resistance to various stresses, easily producing in large scale, and so on) are necessary for cost reduction of biorefinery process. Metagenomics is the study of genetic material recovered directly from environmental samples and it is a powerful tool to identify novel biocatalysts, natural products, and new molecular structures (Iqbal et al. 2012). However, many efforts are required for selecting the target genes because environmental genome has extremely broad diversity. Therefore, enrichment of an environmental genome may make it possible to select the purpose gene efficiently.

In present study, metagenomic libraries were prepared using DNA from raw environmental samples or short enrichment cultures, and screening was based on enzyme activity. The Japanese cedar, *Cryptomeria japonica*, is a widely distributed coniferous tree that is an important plantation tree in Japan. Therefore, cellulase genes that were able to degrade the cellulose of raw cedar wood were main target in this study. In addition, for construction of the effective screening method, the metagenomic libraries were constructed from enrichment bacterial cultures using a different cellulosic carbon source, and the enrichment effect was evaluated.

## Results

Total DNA from the original soil sample (collected from under the pile of cedar wood sawdust) and its enrichment cultures (1st and 2nd enrichment culture with Avicel or unbleached kraft pulp as the carbon source) was extracted, and the 16S rRNA genes were amplified. The purified rRNA gene fragments were cloned into the pGEM-T easy vector. Recombinants were randomly selected, and clonal plasmids were extracted. The sequences of a few bacterial 16S rRNA gene clones (500–600 bp) were determined. A total of 16 clones (4 clones from the original soil [GenBank/EMBL/DDBJ under accession numbers (AN): AB921992 ~ AB921995], 8 clones from 1st enrichment culture with Avicel [AN: AB21996 ~ AB22003], and 4 clones from 2nd enrichment culture with Avicel [AN: AB22004 ~ AB22007]) were analyzed to estimate the bacterial diversity in the original soil and Avicel enrichment cultures. The genes from the original soil bacteria were from several bacterial groups. However, 5 of 8 sequences from the 1st Avicel enrichment culture and all 4 clones from the 2nd Avicel enrichment culture were within the gamma-proteobacteria group. It was expected that the diversity of bacteria has lowered considerably by 2nd enrichment cultivation. Thus, Enrichment cultivation was stopped at 2nd time enrichment.

Five metagenomic expression libraries were constructed. Each library clone contained a 2–7 kb insert (average 4.3 kb), and 23,000–40,000 clones were screened (Table 1), corresponding to 0.1–0.2 Gb of environmental genome. In the soil, approximately 30,000 colonies were screened for cellulase and xylanase activity without any positive colonies. Four active cellulase clones, p1a1–p1a4, were obtained from the 1st Avicel enrichment culture library (25,000 clones). The p1a2–p1a4 sequences were the same cellulase gene (*C1A2*) and encoded part of an identical genomic sequence (*1A2*). Two cellulase genes *C1A1* and *C1A2* contained in respective sequence *1A1* (AN: AB92208) and *1A2* (AN: AB922009) were identified from 1st Avicel enrichment culture. Four active cellulase clones, p2a1–p2a4 were isolated from the 2nd Avicel enrichment culture library (32,000 clones). The sequences of p2a2 and p2a4 were the *1A2* gene. Clone p2a1, and p2a3 have independent cellulase (named *C2A1*, and *C2A3*) within geomic sequence *2A1*(AN: AB9220010) and *2A3* (AN: AB9220011), respectively. Although no active clone was obtained from the 1st pulp enrichment culture, an active cellulase (named C2P3 encoded in genomic sequence *2P3*, AN: AB9220013) and a xylanase (named X2P1 encoded in genomic sequence *2P1*, AN: AB9220012) clone were identified in the 2nd pulp enrichment culture library (40,000 clones).

**Table 1:** **Clones containing glycosyl hydrolase genes from metagenomic libraries constructed from different environmental samples**

Library source	Screening number (Library size)	Active clone		Enzyme name	Sequence name (accession no.)
Soil	30,000 (130 Mb)	n.d.		-	-
Avicel 1st enrichment culture	25,000 (108 Mb)	Cellulase	p1a1	C1A1	*1A1* (AB922008)
		Cellulase	p1a2, p1a3, p1a4	C1A2	*1A2* (AB922009)
Avicel 2nd enrichment culture	32,000 (138 Mb)	Cellulase	p2a1	C2A1	*2A1* (AB922010)
		Cellulase	p2a2, p2a4	C1A2	*1A2* (AB922009)
		Cellulase	p2a3	C2A3	*2A3* (AB922011)
Pulp 1st enrichment culture	23,000 (100 Mb)	n.d.		-	-
Pulp 2nd enrichment culture	40,000 (172 Mb)	Cellulase	p2p3, p2p4, p2p4	C2P3	*2P3* (AB922013)
		Xylanase	p2p1	X2P1	*2P1* (AB922012)

**Figure 1:**
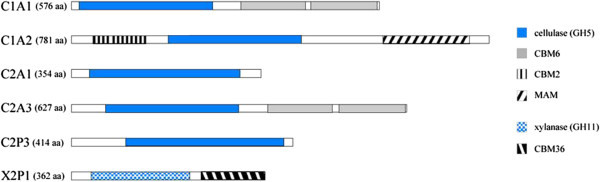
**Schematic diagram of the domain structure of the obtained cellulase genes.**

Sequence analyses of the cloned cellulase and xylanase genes revealed no significant nucleotide homology to known cellulase genes in the databases (Table 2). The best candidates for the *1A1* cellulase gene were cellulase B (622 amino acids) of *Cellvibrio mixtus* (accession AAB61462; 63% identity) and endoglucanase (619 amino acids) of *Hahella chejuensis* KCTC 2396 (accession YP_436051; 62% identity). These homologs have a [glycosyl hydrolase family 5 (GH5)]-[carbohydrate-binding module 6 (CBM6)]-[CBM6] domain structure; therefore, C1A1 is predicted to have a [GH5]-[CBM6]-[CBM6] domain structure (Figure 1). Seven ORFs were identified in the *1A2* sequence (13,375 bp). One ORF (C1A2) had a conserved putative cellulase (endoglucanase)-like sequence with [CBM] and]-[merpin/A5-protein/PTPmu (MAM)] domain (Figure 1). C1A2 had 85% identity to endoglucanase (1,005 amino acids) of bacterium enrichment culture clone CelA10 (accession ACR23656) In *2A1*, a 4136-bp insert and 2 ORFs were identified including an endoglucanase of 354 amino acids (C2A1) which only have a catalytic domain. Sequence *2A3* was an analog of C1A1 with 98.4% similarity at the amino acid level.

**Table 2:** **Sequence analysis and annotation of the genes isolated from the environmental genomic libraries**

Sequence name	Position	Probable function	Most homologous microorganism (%)	Accession no.
*1A1* (3107 bp)	283-2013	Cellulase	*Cellvibrio mixtus* (63%)	AAB61462
	2074-3105	Hypothetical protein	*Azotobacter vinelandii* DJ (82%)	YP_002800613
*1A2* (13375 bp)	763-1539c	Hypothetical protein	Uncultured organism (79%)	ACY24861
	1586-3355c	Hypothetical protein	Uncultured organism (92%)	ACY24860
	3598-4215c	YceI family protein	Uncultured organism (73%)	ACY24860
	5623-7968c	Endoglucanase	bacterium enrichment culture clone CelA10 (85%)	ACR23656
	8137-8745c	Hypothetical protein	Uncultured organism (91%)	ACY24858
	9211-10839c	Cellulose-binding protein	*Cellvibrio japonicus* Ueda107 (60%)	YP_001982938
	11276-12424c	Cellulose-binding protein	*Cellvibrio japonicus* Ueda107 (76%0	YP_001982938
*2A1* (4136 bp)	714-1778c	Endoglucanase	*Sinorhizobium meliloti* (56%)	AAG44364
	1829-3097c	Glycosyl transferase	*Rhizobium etli* CFN 42 (59%)	YP_471521
*2A3* (2539 bp)	530-2413c	Cellulase	*Cellvibrio mixtus* (61%)	AAB61462
*2P3* (7273 bp)	286-957	Glutathione S-transferase-like protein	*Polaromonas* sp. JS666 (72%)	YP_546944
	1081-1947c	Putative protein		
	2584-4932c	Hypothetical protein	*Bacillus cereus* SJ1 (35%)	ZP_07057046
	5249-6493c	Glycoside hydrolase	*Pseudoxanthomonas suwonensis* 11-1 (70%)	YP_004146661
*2P1* (3551 bp)	87-872c	Hypothetical protein	*Paenibacillus dendritiformis* C454 (39%)	ZP_09674415
	1046-2128c	Acetylxylan esterase	*Paenibacillus sp. aloe*-11 (77%)	ZP_09772595
	2192-3280c	Endo-1,4-beta-xylanase	*Paenibacillus sp. aloe*-11 (80%)	ZP_09772596

**Figure 2:**
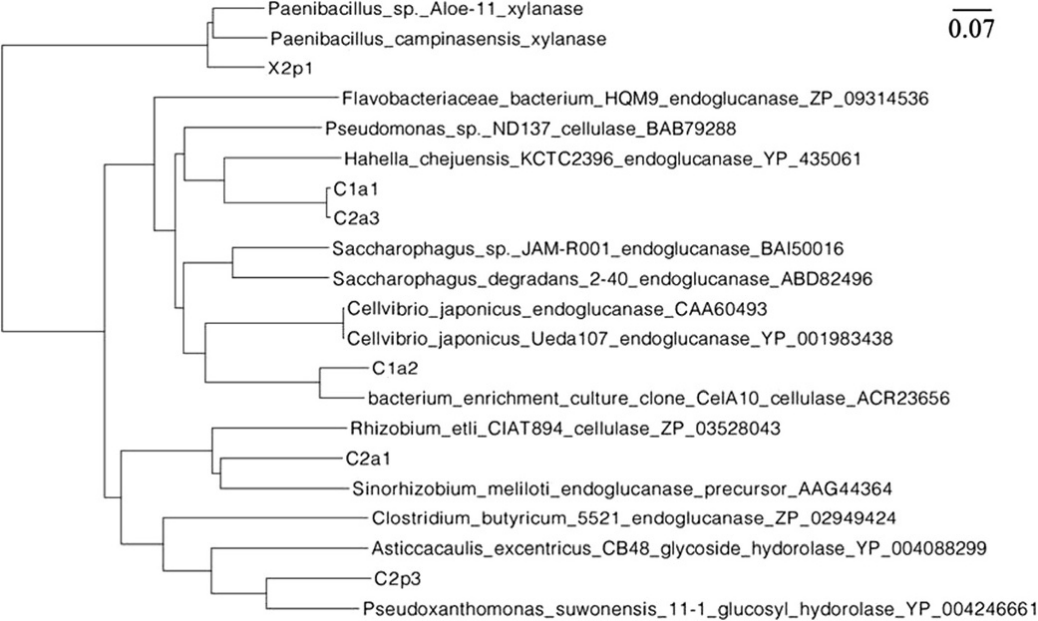
**Unrooted amino acid phylogenetic tree based on an alignment of the isolated cellulases and xylanase with homologous enzymes.** The multialignments were performed in ClustalX, and the phylogenetic tree was constructed by the neighbor-joining method.

Clones 2p3 and 2p1 were obtained from the pulp enrichment cultures. Sequence *2P3* contained a putative glycoside hydrolase gene (*C2P3*) and 3 other ORFs, and sequence *2P1* consisted of a putative endo-1,4-β-xylanase (X2P1) and 2 other ORFs. Cellulase C2P3 (414 amino acids) consisted only of a glycosyl hydrolase domain. Phylogenetic analyses of the isolated cellulases revealed that the isolated glycosyl hydrolases were grouped in four independent branches where they clustered with typical GH family members, while xylanases were branched from the cellulase cluster (Figure 2; see Additional file 1: Figure S1 in the supplemental material for multiple alignment).

Six glycosyl hydrolase genes (5 cellulases and a xylanase) were obtained from enrichment cultures. Three genes (*C1A1*, *C1A2*, and *C2A3*) had one or two CBMs, and their cellulase activity was measured using crystalline cellulose (Avicel) and wood powder (extract-free cedar powder). The three crude cellulases generated reducing sugar from crystalline cellulose (Avicel). In addition, they generated the equivalent level of reducing sugar from extract-free cedar wood powder (Table 3). In contrast, cellulase activity (the production of reducing sugar) of Cellulosin T3 for cedar wood powder was lower than Avicelase activity.

**Table 3:** **Cellulase activity for crystalline cellulose (Avicel) and cedar wood powder**

Cellulase name	Production of reducing sugar (μg/ml)		Relative activity (%) (sugi/avicel)
	Avicel	Ceder wood powder	
C1A1	200.2 (±11.3)	217.4 (±4.9)	108.6
C1A2	195.5 (±4.4)	203.6 (±6.9)	104.2
C2A3	197.7 (±39.1)	175.1 (±8.3)	88.5
Cellulosin T3	328.7 (±26.0)	67.7 (±12.9)	20.0

## Discussion

It was reviewed that a number of cellulase genes had been isolated from metagenomic libraries (Duan and Feng 2010; Li et al. 2009), the hit rate was too low, at least 100 Mb metagenomic per one cellulase gene is required by functional screening of the library from soil or sediment sample. On the other hand, one cellulase gene was provided per 10-50 Mb in the metagenomic library made from rumen of cow, buffalo etc. In the report of the screening from library made from enrichment culture, one cellulase per 40 Mb was provided in the case of highest hit rate (Voget et al. 2003). In present study, the size of the metagenomic library that was constructed from soil was around 130 Mb; however, no positive cellulase/xylanase clones were obtained (Table 1). On the other hand, the hit rate was about 1 cellulase gene per 54 and 46 Mb metagenomic DNA from 1st and 2nd enrichment cultures with Avicel, respectively. The cause that the number of isolated cellulase genes after 2nd avicel enrichment only slightly increased was predicted that the bacterial diversity in enrichment culture has considerably reduced already by 1st Avicel enrichment, and that the diversity of cellulase-producing bacteria did not change so much between 1st and 2nd Avicel enrichment. Because that an identical cellulase gene (*C1A2*) has been obtained from metagenomic libraries of 1st and 2nd enrichment culture. When unbleached pulp was used as a carbon source for enrichment, glycoside hydrolase genes (cellulase and xylanase) were provided with hit rate of 0/100 Mb (1st enrichment) and 2/172 Mb (2nd enrichment). Although the screening efficiency improved by repeating enrichment, the efficiency was greatly different between enrichment cultures using Avicel and unbleached pulp. It was presumed that non-cellulolytic bacteria have grown vigorously in enrichment culture with unbleached pulp than enrichment culture with Avicel, because unbleached pulp has more various components that are easy to utilize such as amorphous cellulose and hemicellulose than avicel as crystaline cellulose. The efficiency of screening was improved by repeating enrichment and was also possible to improve more than three times by enrichment cultivation with strong selective pressure. Because the 16S rRNA gene diversity suggested that bacterial diversity decreased by the enrichment and the positive cellulase clones contained the same cellulase gene were identified from 1st and 2nd enrichment culture with Avicel, repeating the enrichment cultivation reduced the cellulase gene diversity (Table 1). However, it seems that the cellulase genes identified in this study had been shown enough diversity (Figure 2).

Previously, most purified cellulases from metagenomic library showed no/weak activity toward crystalline cellulose (Duan and Feng 2010). In this study, the cellulases containing a CBM hydrolyzed Avicel and had cellulase activity against cedar wood powder (Table 3), because the soil used in this study was collected from cedar sawdust sediment. Generally, cellulases do not show sufficient activity against lignocellulosic material, because hemicelluloses and lignin work as physical barriers and prevent the access of enzymes to cellulose surface. In addition, soluble hemicelluloses may strongly inhibit the cellulase activity and lignin adsorbed enzymes non-specificity. (Rahikainen et al. 2013a; Zhang et al. 2012). Because Cellulosin T3 has the xylanase activity, there is a possibility that soluble xylan was produced and has inhibited cellulase activity during process. Research group of Rahikainen et al has studied about detail of inhibition of cellulases by lignin. They reported that the lignin-rich residue provided by enzymatic hydrolysis of Spruce was found to have a strong inhibitory effect on enzymatic hydrolysis of microcrystalline cellulose (MCC). Inhibition of cellulase activity by lignin became strong depending on temperature, and endoglucanase activity of lignin-bound commercial *T. reesei* cellulase mixture was lost ~ 40% at 45°C for 1.5 hours (Rahikainen et al. 2011). And it was mentioned that the lignin-binding properties were different depending on CBM type and the surface properties of catalytic domain. (Rahikainen et al. 2013b). In this study, however, crude cellulases showed comparable activity against crystalline cellulose and cedar wood flour. It is possible that the structures of these enzymes do not adsorb to the lignin surface or that other crude proteins are adsorbed to lignin, protecting the cellulase activity.

Although cellulases C1A1 and C2A3, which have tandem CBM6 domains (Figure 1), has similar domain construction of endoglucanase of *Celivibrio mixtus* (endoglucanase 5A) has been investigated (Henshaw et al. 2004; Pires et al. 2004). The family 6 CBM from endoglucanase 5A also has two binding sites. The binding site in ‘cleft A’ can accommodate the chain ends of β-1,4-glucans, β-1,3-glucans and xylans. ‘Cleft B’ binds to internal regions of β-1,4-glucans and mixed β-(1,4) (1,3)-glucans. In addition, Kimura and Kamei investigated β-1,3(4)-glucanase A (GluA) of marine bacterium *Pseudomonas* sp. PE2 also has tandem CMB6 domains, suggesting that the tandem CBM of GluA may play a key role in the binding of Avicel and xylan and is very important for binding insoluble polysaccharides (Kitamura and Kamei 2006). These observations suggest that tandem-repeated CBM of cellulases C1A1 and C2A3 may play key role in binding and hydrolyzing crystalline cellulose and other insoluble polysaccharides in lignocellulosic materials. Cellulase C1A2 had a putative MAM domain (Figure 1). The MAM domain is an extracellular domain that mediates protein-protein interactions and is found in a diverse set of proteins that function in cell adhesion (Beckmann and Bork 1993). However, it is still unknown how the MAM-like domain works in hydrolysis of lignocellulose material. Although these cellulases isolated in present study are analog domain structure of known cellulases, the understanding of details of the function of each domain for hydrolysis of lignocellulose is not enough. Therefore, it is necessary to clarify the characteristics of these cellulases in future studies.

## Conclusions

In this study, metagenomic libraries were constructed from enrichment cultures that were seeded with the soil that was covered to cedar sawdust to obtain cellulases that degrade crystalline cellulose. The cellulase genes were identified effectively by using enrichment cultivation, and three of cellulase active clones showed activity against crystalline cellulose and cedar wood powder. These results demonstrated that this technique is a powerful tool for obtaining cellulases that have activity toward crystalline cellulose.

## Methods

### Bacterial strains, sample sites, and enrichment cultures

*E. coli* DH5α was the host and the plasmid pUC119 (TaKaRa Bio.) was the vector for cloning experiments.

To construct metagenomic libraries, a soil sample under *C. japonica* sawdust sediment was collected from a backyard, in Kyushu University, Fukuoka, Japan. Enrichment was performed in modified M9 minimal medium with microcrystalline cellulose (Avicel^®^) or unbleached hardwood kraft pulp as a carbon source. Enrichment cultures from the soil sample were prepared by adding 1.0 g soil to 40 mL sterile minimal medium containing 20 g/L carbon source, 6.0 g/L Na_2_HPO_4_, 3.0 g/L KH_2_PO_4_, 0.5 g/L NaCl, 1.0 g/L Yeast Extract (Difco), 1 mM MgSO_4_, 0.1 mM CaCl_2_, and 0.04 mM FeSO_4_. Cultures were grown at 37°C for 7 days at 120 rpm (1st enrichment culture). Then, 0.5 mL culture medium was transferred to new minimal medium and incubated for an additional 7 days (2nd enrichment culture). Bacteria were recovered from each enrichment culture by centrifugation (3,000 × *g* for 20 min), and the obtained pellets were washed with 50 mM Tris-HCl (pH 8.0). The resulting precipitates were used for DNA extraction.

### Total DNA extraction

DNA was extracted using a protocol based on the direct lysis method of Zhou et al. (1996) with minor modifications. Briefly, collected samples (5 g) were extracted by adding 13.5 mL DNA extraction buffer (100 mM Tris-HCl pH 8.0, 100 mM EDTA, 100 mM sodium phosphate, 1.5 M NaCl, and 1% cetyltrimethylammonium bromide) and 0.1 mL proteinase K (100 mg/mL) with horizontal shaking for 30 min at 37°C. Next, 1.5 mL 20% (w/v) sodium dodecyl sulfate was added, and the suspensions were incubated at 65°C for 2 h with gentle shaking every 15–20 min before the supernatant was collected by centrifugation at 3000 × *g* for 10 min. The pellet was re-extracted, and the combined supernatants were extracted with an equal volume of phenol-chloroform (1:1, v/v). The aqueous phase was recovered by centrifugation, and the DNA was precipitated with 0.6 volume of isopropanol at room temperature for 20 min. A pellet of crude nucleic acids was obtained by centrifugation at 12,000 × *g* for 10 min. After washing with 70% (v/v) ethanol, the DNA was re-suspended in 1 mL 10 mM Tris-HCl (pH 8.5) and purified by electrophoresis in a 1.0% low melting point agarose gel (agarose L, Wako). Then, DNA fragments over 22 kb were collected and extracted. Recovered DNA was precipitated with an equal volume of isopropanol, washed with 70% ethanol, and air-dried. Dried DNA was dissolved with 50 μL TE buffer (10 mM Tris-HCl, 1 mM EDTA, pH 8.0).

Purified DNA samples were used to construct genomic DNA libraries following partial digest with Sau3A1. Digested DNA was roughly fractionated by polyethylene glycol precipitation with 15 mM MgCl_2_ and 7.5% polyethylene glycol 6000. The DNA fragments were ligated into the pUC19 vector, which had been digested with BamHI and phosphatase treated with the TaKaRa Ligation Kit v2.1. *E. coli* DH5α was transformed with ligated pUC19, and transformants were selected on Luria-Bertani medium (LB) agar plates containing ampicillin (100 μg/mL) and 0.2% (w/v) rimazol brilliant blue dyed CMC or xylan (Kok and van der Velde 1991). By velvet replication (Lederberg and Lederberg 1952), the library was provided for both active screening. The active cellulolytic clones were visible by a clear halo against a blue background. Plasmid DNA was isolated from positive cellulolytic clones, and DNA was sequenced at Macrogen Japan, Tokyo University of Agriculture, using ABI BigDye Terminator v3.1 Cycle Sequencing Kits and an ABI 3730xl Analyzer. Complete coverage of the sequence was obtained by primer walking from the 5′ and 3′ ends or the shotgun-based approach described by Emonet et al. (2007). Sequences were compared to those in databases. The open reading frame (ORF) finder from NCBI was used to identify possible ORFs.

### Cloning and DNA sequencing of the 16S rRNA gene

PCR amplification of the 16S rRNA genes was performed using purified metagenomic DNA as a template and bacterial universal primers (Lane 1991). The PCR conditions were as follows: initial denaturation at 95°C for 9 min and 18 cycles of 95°C for 1 min, 50°C for 1 min, and 72°C for 2 min. PCR products were ligated into the pGEM-T easy vector (Promega) and transformed into competent *E. coli* DH5α cells. Clones were randomly selected and sequenced. Sequencing was performed with primer 907R (5′-CCGYCAATTCMTTTRAGTTT-3′). All sequences (500–600 nucleotides) were compared with those in the GenBank database (http://www.ncbi.nlm.nih.gov/BLAST) using BLAST. Multiple sequence alignment was carried out using ClustalX software, and a phylogenic tree was generated by the neighbor-joining method (Saitou and Nei 1987).

### Cellulase activity assays

Cellular extracts were prepared by dissolving positive cellulolytic *E. coli* clones with bugbuster^®^ (Merck). The supernatants were concentrated 7.5 times by ultrafiltration. To measure the cellulase activity, crude concentrates were incubated with 1.0% (w/v) Avicel or cedar wood powder (extract free) in 100 mM Tris-HCl (pH 6.8) for 2 h at 50°C. The released reducing sugars were measured as D-glucose equivalents (Miller 1959). Cellulosin T3 (HBI Enzyme Inc., Hyogo, Japan) was used as a positive control.

## Electronic supplementary material

Additional file 1: Figure S1: Multiple amino acid sequence of the isolated C1A1, C1A2, C2A1, C2A3, and C2P3 with their close relatives as identified by BlastP. (DOCX 637 KB)
