# Supplementary material for: Identification of novel glycosyl hydrolases with cellulolytic activity against crystalline cellulose from metagenomic libraries constructed from bacterial enrichment cultures
Source: Springerplus. 2014 Jul 16;3:365. doi: 10.1186/2193-1801-3-365 (PMC4112031; doi:10.1186/2193-1801-3-365)
Supplement: Supplementary file 1 [file 40064_2014_1064_MOESM1_ESM.docx]

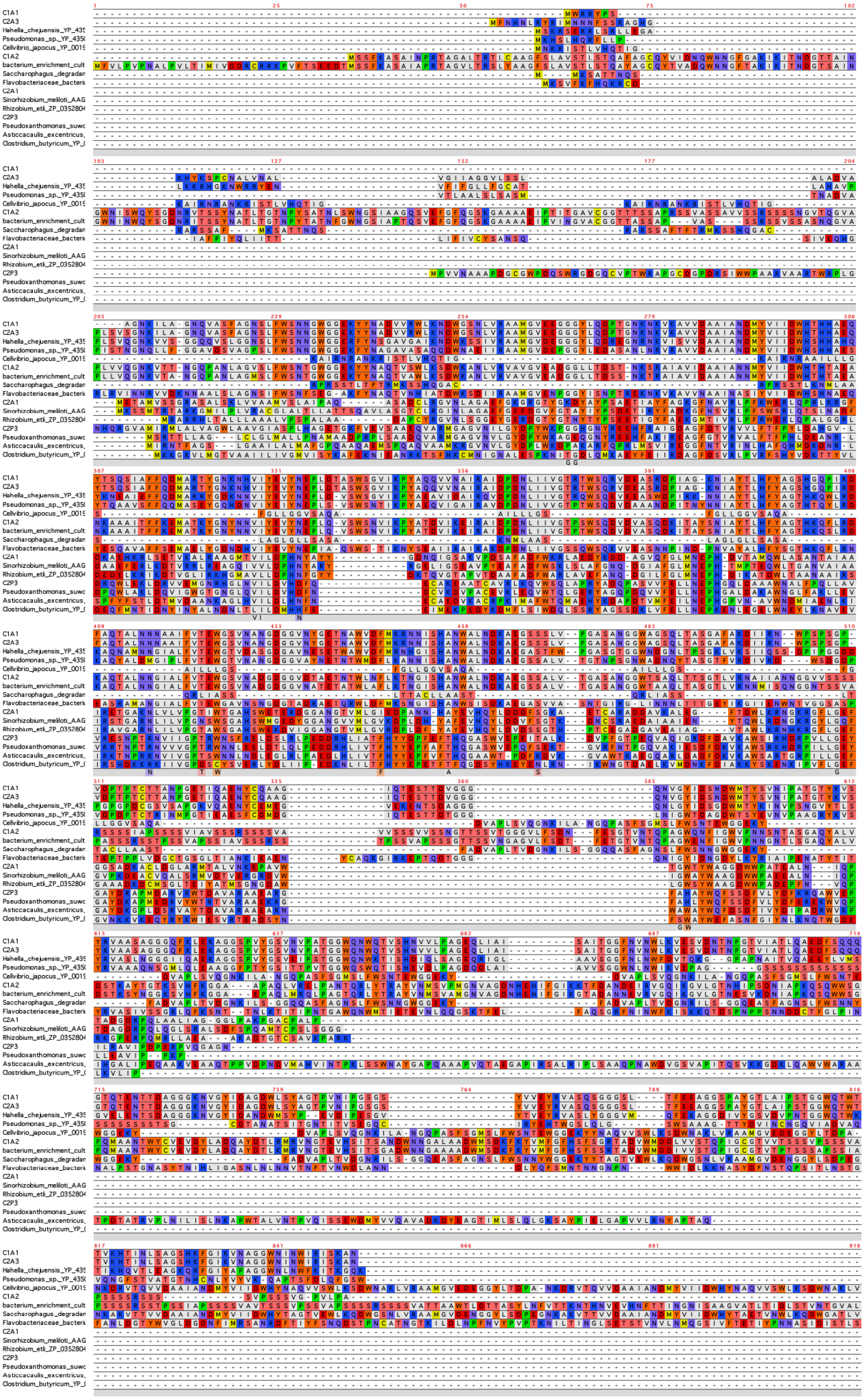
C1A1
C2A3
Hahella_chejuensis_YP_435
Pseudomonas_sp._YP_435(
Cellvibrio_japocus_YP_0019
C1A2
bacterium_enrichment_cult
Saccharophagus_degradan
Flavobacteriaceae_bacteri
C2A1
Sinorhizobium_meliloti_AAG
Rhizobium_etli_ZP_0352804
C2P3
Pseudoxanthomonas_suwc
Asticcacaulis_excentricus_
Clostridium_butyricum_YP_(
